# Androgen receptors are acquired by healthy postmenopausal endometrial epithelium and their subsequent loss in endometrial cancer is associated with poor survival

**DOI:** 10.1038/bjc.2016.16

**Published:** 2016-03-01

**Authors:** A M Kamal, J N Bulmer, S B DeCruze, H F Stringfellow, P Martin-Hirsch, D K Hapangama

**Affiliations:** 1 Department of Women's and Children's Health, Institute of Translational Medicine, University of Liverpool, Liverpool L8 7SS, UK; 2 The National Center for Early Detection of Cancer, Oncology Teaching Hospital, Baghdad Medical City, Baghdad, Iraq; 3 Institute of Cellular Medicine, Newcastle University, Newcastle upon Tyne NE2 4HH, UK; 4 Liverpool Women's Hospital NHS Foundation Trust, Liverpool L8 7SS, UK; 5 Lancashire Teaching Hospital NHS Trust, Lancaster University, Preston PR2 9HT UK

**Keywords:** androgen receptor (AR), endometrial cancer, postmenopausal endometrium, metastatic lesions, ER*β*, ER*α*, PR, outcome

## Abstract

**Background:**

Endometrial cancer (EC) is a hormone-driven disease, and androgen receptor (AR) expression in high-grade EC (HGEC) and metastatic EC has not yet been described.

**Methods:**

The expression pattern and prognostic value of AR in relation to oestrogen (ER*α* and ER*β*) and progesterone (PR) receptors, and the proliferation marker Ki67 in all EC subtypes (*n*=85) were compared with that of healthy and hyperplastic endometrium, using immunohistochemisty and qPCR.

**Results:**

Compared with proliferative endometrium, postmenopausal endometrtial epithelium showed significantly higher expression of AR (*P<*0.001) and ER*α* (*P*=0.035), which persisted in hyperplastic epithelium and in low-grade EC (LGEC). High-grade EC showed a significant loss of AR (*P<*0.0001), PR (*P<*0.0001) and ER*β* (*P<*0.035) compared with LGEC, whilst maintaining weak to moderate ER*α*. Unlike PR, AR expression in metastatic lesions was significantly (*P*=0.039) higher than that in primary tumours. Androgen receptor expression correlated with favourable clinicopathological features and a lower proliferation index. Loss of AR, with/without the loss of PR was associated with a significantly lower disease-free survival (*P<*0.0001, *P<*0.0001, respectively).

**Conclusions:**

Postmenopausal endometrial epithelium acquires AR whilst preserving other steroid hormone receptors. Loss of AR, PR with retention of ER*α* and ER*β* may promote the unrestrained growth of HGEC. Androgen receptor may therefore be a clinically relevant prognostic indicator and a potential therapeutic target in EC.

Endometrial cancer (EC) is the commonest gynaecological malignancy in the developed world, with an increasing incidence related to obesity (Cancer Research UK, 2013; Lindemann *et al*, 2010). Traditional classifications of EC are based either on clinical and endocrine features (e.g., types I and II; Bokhman, 1983) or on histopathological characteristics (e.g., endometrioid, serous and clear-cell carcinomas, and carcinosaroma; Murali *et al*, 2014). More recently, a molecular classifier that can be combined with clinicopathological features to predict outcome has also been proposed (Kandoth *et al*, 2013; Talhouk *et al*, 2015) In contrast with previous reports, recent data suggest that type I and II ECs may share common aetiological factors, including their response to/stimulation by oestrogen and other ovarian steroid hormones (Setiawan *et al*, 2013).

The endometrium is the main target organ for ovarian hormones, and steroid hormones are implicated in carcinogenesis in endometrium and other classical hormone-responsive tissues such as breast and prostate. Endometrial cancer is generally a disease of the postmenopausal (PM) period that is defined by cessation of the cyclical production of ovarian hormones. As these hormones exert their effect via their cognate receptors, in common with other hormone-responsive cancers such as breast cancer (Tagnon, 1977; Thike *et al*, 2001), the hormone receptor status of ECs would be expected to have a role in predicting clinical outcome and guiding therapeutic choice (Zhang *et al*, 2015). Unlike breast cancer (Breast Cancer Consensus C *et al*, 1994), steroid receptor status is not routinely reported for EC, yet increasingly clinical oncologists in the UK seek this information in high-grade EC to make therapeutic decisions beyond standard surgery (Singh *et al*, 2007).

Defining the contribution of all steroid receptors (including AR, which has not been described in detail in EC previously), to the initiation, progression and prognosis of EC will improve the understanding of the hormonal changes that precede and potentially drive EC tumorigenesis.

In this study, the spatial and temporal expression pattern of AR in normal and hyperplastic PM endometrium was compared with that of healthy proliferative phase (PP) endometrium. The expression profile of AR in EC was investigated and compared with endometrium from healthy PM women. Expression of AR, ER*α*, ER*β*, PR and the proliferation marker Ki67 was correlated in normal (premenopausal PP and PM), hyperplastic and malignant endometrium to gain a better insight in to the interplay between oestrogens, progesterone and androgens in healthy and neoplastic endometrium. Finally, the prognostic value of AR and other sex hormone receptors in EC was evaluated.

## Materials and Methods

### Patient population

Patient groups are detailed in Table 1. A total of 85 EC, 16 metastatic lesions (3 lymph node, 7 soft tissue, 3 parametrium and 3 omentum), 12 hyperplastic (4 without cytological atypia, EHNA; 8 with cytological atypia, EHA) and 28 full-thickness normal endometrial biopsies were collected from patients undergoing hysterectomy in Liverpool Women's Hospital and Lancashire Teaching Hospitals Trusts from 2009 to 2014. The study was approved by Liverpool and Cambridge Adult Research Ethics Committee (LREC 09/H1005/55, 11/H1005/4 and CREC 10/H0308/75). The histological type and grade of EC specimens were assigned by experienced gynaecological pathologists according to the International Federation of Gynecology and Obstetrics (FIGO; Zaino *et al*, 1995). Endometrial cancer was categorised as low-grade (LGEC, including grade 1 and grade 2 endometrioid EC) or high-grade tumours (HGEC, including grade 3 endometrioid, serous and clear-cell carcinomas, and carcinosarcoma) (Voss *et al*, 2012) for subsequent analysis of immunohistochemistry (IHC) data (Table 1). Proliferative phase specimens were assigned according to last menstrual date and histological criteria (Noyes *et al*, 1950; Dallenbach-Hellweg *et al*, 2010). All samples were split in to two; one was fixed (⩾24 h in 4% (v/v) buffered formalin) and paraffin-embedded for immunohistochemical staining, and the other part was immediately placed into RNA*later* (Sigma, Dorset, UK) for RNA extraction for PCR.

Patient clinicopathological and demographic details were retrieved by review of hospital notes and clinical databases. None of the patients received hormonal treatments, chemotherapy or pelvic radiation before surgery.

### Immunohistochemistry

After antigen retrieval at pH6 as previously described (Hapangama *et al*, 2012) 3 *μ*m formalin-fixed paraffin-embedded tissue sections were immunostained with anti-human steroid receptor antibodies and Ki67; antibody sources, concentrations and incubation conditions are detailed in Supplementary Table 1. Detection was with the ImmPRESS polymer-based system and visualisation was with ImmPACT DAB (Vector Laboratories, Peterborough, UK) used in accordance with the manufacturer's instructions. Sections were lightly counterstained in Gill 2 Haematoxylin (Thermo Scientific, Runcorn, UK), dehydrated, cleared and mounted in synthetic resin. Matching isotype (0.5 *μ*g ml) replaced the primary antibody as a negative control, with internal positive controls performed in each staining run.

### Analysis of IHC staining

Immunostaining for the four steroid receptors was assessed semi-quantitatively using a four-tiered Liverpool endometrial steroid quick score (LESQS). The final score out of 12 was calculated by multiplying the proportion of positive cells (1–10%=1, 11–20%=2, 21–40%=3 and >40%=4) by the staining intensity categories (0=no staining, 1=weak, 2=moderate and 3=strong). The detailed description, optimisation and validation of this scoring system are presented in Supplementary Methods. The Ki67 proliferative index (PI) was evaluated as the percentage of immunopositive cells, of any intensity. The entire section was evaluated at × 400 magnification as previously described (Al Kushi *et al*, 2002).

Epithelial and stromal cell staining was scored separately in PM, and malignant endometrium and stratum basalis of healthy PP endometrium by two independent observers (AMK and DKH). Discrepencies between the two observers were resolved by re-evaluating the samples together and agreeing on a final score.

For the purpose of description, scores 1–4 were considered as low, 5–8 as moderate and 9–12 as high levels of expression. For survival analysis and correlation with clinicopathological analysis, data were subclassified as immunopositive when >10% of the neoplastic cells expressed the target protein at any intensity (LESQS ⩾2) and immunonegative if <2 (Pertschuk *et al*, 1996).

### RT–qPCR

Total RNA from tissue samples was extracted using TRIzol Plus RNA Purification System (Life Technologies, Paisley, UK), and quantified by NanoDrop ND-1000 (Thermo Fisher Scientific, Loughborough, UK). Total RNA was reverse transcribed using AMV First Strand cDNA synthesis kit (New England Bio Labs, Hertfordshire, UK) after DNase treatment (DNase I (#M0303), New England Bio Labs, Hertfordshire, UK), using the manufacturer's protocol. cDNA was amplified by qPCR using JumpStart SYBR Green supermix (Sigma, Dorset, UK) and the Light Cycler 96 Roche Real-Time System (Roche Diagnostics Ltd. BurgessHill, UK). Primers are listed in Supplementary Table 2. Relative transcript expression was calculated by the ΔΔCT method, normalised to the reference gene *YWHAZ* (Sadek *et al*, 2011) using Biogazelle qbase+ software (Biogazelle NV, Zwijnaarde, Belgium)

### Statistical analysis

Statistical differences between groups were calculated by non-parametric tests (Kruskal–Wallis and/or Mann–Whitney *U*-test or Wilcoxon signed-rank test) using Statistical Package for the Social Sciences (SPSS) version 21 (IBM Corp, Armonk, NY, USA). Descriptive values were presented as median and range. The correlations between immunoexpression scores were examined with Spearman test, and association between immunoscores and the multiple clinicopathological parameters with Pearson *χ*^2^. Disease-free survival (DFS) was calculated from the date of surgery to the date of recurrence, death or the date on which the patient was last seen. For survival analysis each parameter was categorised, and survival curves were obtained using the Kaplan–Meier method. The Cox proportional hazards regression model was used to identify the independent prognostic factors. Only variables with *P*<0.05 in the univariate analysis were included in the multivariate model. *P*<0.05 was considered significant.

## Results

### Demographic data

Patient demographics are detailed in Table 1. Women with HGEC were significantly older than those with LGEC (*P<*0.0001). Patients with EHA were significantly younger than those with LGEC (*P*=0.016). Healthy PM controls had the lowest BMI compared with women who had EHA (*P*=0.007) and LGEC (*P*=0.022). There was no significant difference in BMI between LGEC and HGEC.

### IHC analysis of AR and other steroid receptor expression

**Figure 1:**
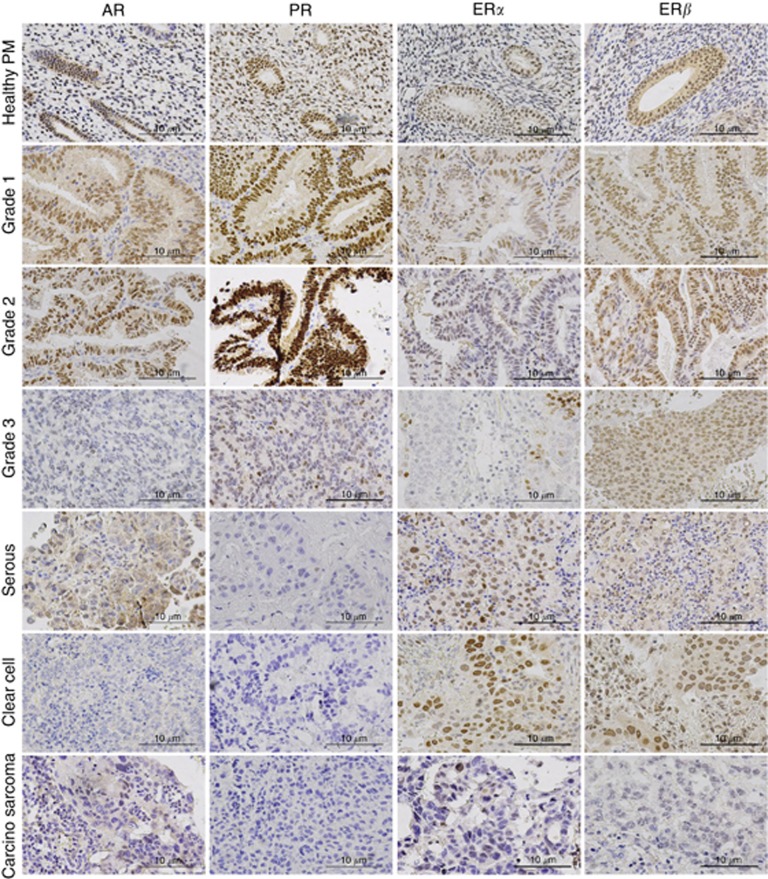
**Representative photomicrographs of AR, PR, ER*α* and ER*β* in human endometrium.** Healthy PM endometrium, grade 1–3 endometrioid carcinoma, serous carcinoma, clear-cell carcinoma and carcinosarcoma were immunostained for AR, PR, ER*α* and ER*β*. Positive staining appears brown. Magnification × 400.

All four steroid receptors were expressed by the endometrium (Figure 1). The main focus of interest, AR, was expressed by both the epithelium and stroma. At a subcellular level, both cytoplasmic and nuclear AR staining was observed; only nuclear immunostaining suggesting transcriptionally active AR with functional relevance was scored and semi-quantified.

### Healthy PM endometrial epithelial cells aquire AR and preserve ERs and PR

**Figure 2:**
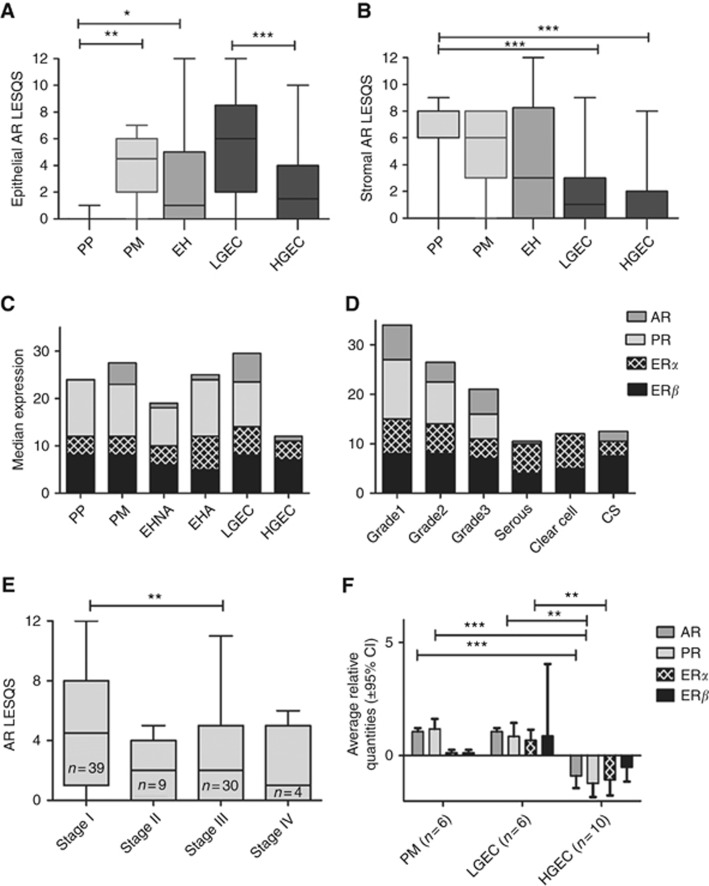
**Protein and mRNA expression levels for Steroid receptors in endometrial cancer.** (**A**) Expression of AR in the epithelium and (**B**) stroma of healthy PP, PM, endometrial hyperplasia (EH), LGEC and HGEC. (**C**, **D**) Stacked graphs represent the median expression of AR, PR, ER*α* and ER*β* in (**C**) PP, PM, endometrial hyperplasia no atypia (EHNA), endometrial hyperplasia with atypia (EHA), LGEC and HGEC; (**D**) endometrial cancer subtypes, including carcinosarcoma (CS). (**E**) Expression of AR protein according to endometrial cancer stage. (**F**) Steroid receptor mRNA levels in human endometrial tissue, PM, LGEC and HGEC. *P<0.05, **P<0.001, ***P<0.0001.

The dominant steroid receptor in both epithelium and stroma of the healthy endometrium was PR. In PP endometrium, AR expression was largely limited to stromal cells in both the stratum basalis and functionalis with absent epithelial AR staining (Supplementary Figure 1a). In contrast, the most striking feature of non-proliferating PM endometrium was the emergence of nuclear AR immunopositivity (*P<*0.001) in the epithelial cells (Figure 2A). Compared with epithelial cells in PP stratum basalis, PM epithelial cells also expressed significantly higher levels of ER*α* (*P*=0.035) (Figure 2C and Supplementary Table 3); however, there were no differences in ER*β* and PR expression scores or in the ER*α*/ER*β* ratio. The stromal expression scores for both PM and PP stratum basalis were similar for all the steroid receptors examined (Supplementary Table 3).

### AR expression and ER*α*/ER*β* ratio is increased in atypical endometrial hyperplasia

PR expression was the strongest of the steroid receptors in both epithelial and stromal compartments of EH (Supplementary Table 3). Interestingly, compared with PP epithelium, nuclear AR expression was significantly higher in the epithelial cells in EHA (*P*=0.025), but no significant change was observed when compared with PM epithelium (Figures 2A and C). Endometrial stroma in EHA showed significant loss of AR expression compared with PP stroma (*P<*0.0001). The general trend of ER*α* epithelial expression in EHA was higher than that of PP (*P*=0.076) and PM (*P*=0.547). In contrast, the trend of ER*β* expression in EHA was lower than that of PP (*P*=0.128) and significantly lower compared with PM endometrium (*P*=0.014, Figure 2C). Thus, ER*α*/ER*β* in EHA was generally higher than that of normal endometrium; this was significant compared with PP (*P*=0.041).

In common with AR, both ER*α* (*P*=0.043) and ER*β* (*P*=0.045) expression scores were significantly decreased, whereas PR was increased (*P*=0.02) in the stroma of EHA compared with PM endometrium. Epithelial PR expression levels were preserved at a level that was comparable to that of healthy PP and PM samples. The PI, assessed by Ki67 immunopositivity, of epithelial cells in EHNA and EHA was similar to that of PP epithelium but, as expected, was significantly higher than PM epithelium (EHNA *P*=0.016, EHA *P*=0.017, Supplementary Figure 1b).

### AR, PR and ER*β* are downregulated in HGEC

We chose healthy PM tissue as the healthy comparator for steroid receptor expression scores of EC samples. ER*β* was the predominant steroid receptor expressed in both LGEC and HGEC (Figures 1 and 2C). AR (*P*=0.10, Figures 1 and 2A and C) and ER*α* (*P*=0.05, Figure 2C) staining scores showed a trend to being increased in LGEC, with simultaneous reduction in PR (*P*=0.08, Figure 2C). This was associated with a significant reduction in stromal expression of AR (*P<*0.0001, Figure 2B), ER*α* (*P<*0.0001) and PR (*P<*0.0001) when compared with healthy PM controls. There was no significant change in ER*β* scores (stromal and epithelial) of LGECs compared with healthy controls, although the ER*α*/ER*β* ratio was higher in LGEC compared with PP (*P*<0.0001); and PM (*P*=0.02).

The epithelial cells of HGEC and the surrounding stroma showed a general reduction in the expression of all four steroid receptors compared with healthy PM tissue (Supplementary Table 3). Compared with LGEC, in HGEC epithelial AR (*P<*0.0001), PR (<0.0001) and ER*β* (*P*=0.035) scores were significantly lower (Figures 1 and 2A and C). Within the subtypes of HGEC, the most pronounced loss of AR was observed in the clear-cell carcinoma group (*P*=0.001); albeit ER*α* expression scores in the same group remained moderate to strong (Figure 2D). Weak to moderate cytoplasmic AR was observed in 33% (11 out of 33) HGEC in the absence of nuclear staining (Figure 1, serous). The expression scores for both PR (*P*<0.0001) and ER*β* (*P*=0.003) in HGEC epithelial cells and surrounding stroma PR (*P*<0.0001) and ER*β* (*P*=0.02; Figure 2C) were significantly lower than healthy PM counterparts. Interestingly, in non-endometrioid HGEC, 26 out of 33 (78.8%) showed loss of PR whilst all were ER*β*+, and 30 out of 33 (90.9%) were also ER*α*+. Progesterone receptor loss was limited to only 5 out of 15 (33.3%) of the endometrioid HGEC. Expression of AR in non endometrioid HGEC was comparable to PR; 16 out of 33 (48.5%) were AR-negative, and 13 of these were also negative for PR. Furthermore, only AR expression (not PR) reduced significantly with advanced FIGO stage (stage I *vs* stage III, *P*=0.006, Figure 2E).

### Metastatic lesions aquire AR

**Figure 3:**
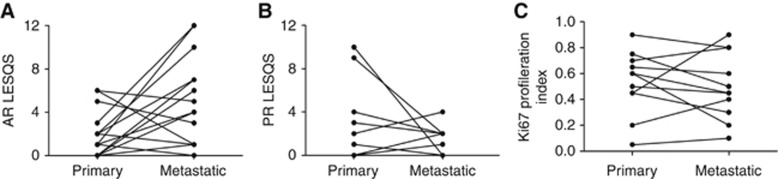
**Immuno-expression scores of AR, PR and Ki67 in metastatic compared with matched primary tumours.** (**A**) AR (**B**) PR and (**C**) Ki67.

Nuclear AR was observed in 10 out of 16 (62.5%) (Figure 3A) metastatic lesions, and expression scores were significantly higher compared with the matched primary lesions (*P*=0.03). By contrast, only 6 out of 15 (40%) (Figure 3B) of metastatic lesions expressed PR. Although the median expression of Ki67 was generally lower in metastatic lesions (45%) than in the matched primary tumour (60%) (Supplementary Figure 1b; E and F), the difference in the expression pattern of both PR and Ki67 between the two groups was not statistically significant (Figure 3C).

### AR expression positively associates with favourable prognostic factors

Endometrial cancer epithelial AR correlated positively with PR (*r*=0.63, *P<*0.0001), whilst there was a negative correlation with Ki67 (*r*=−0.43, *P*= 0.0004, Supplementary Table 4). When expression of steroid receptors (as positive or negative) was correlated with each clinicopathological parameter, both AR and PR expression correlated positively with well-differentiated tumours and those without cervical invasion, yet only AR expression showed positive correlation with early FIGO stages (*P*=0.048, Supplementary Table 5). Intriguingly, concurrent loss of AR and PR showed a significant positive correlation with higher tumour grades (*P<*0.0001), late FIGO stages (*P*=0.004), deep myometrial invasion (*P*=0.003), extrauterine invasion (*P*=0.048) and cervical invasion (*P<*0.0001). ER*α* did not show a significant correlation with clinicopathological parameters; however, a high ER*α*/ER*β* ratio was associated with invasion of the cervical stroma (*P*=0.041) and showed a trend to be associated with advanced stage tumours (*P*= 0.057).

### Loss of AR adversely influences patient outcome

**Figure 4:**
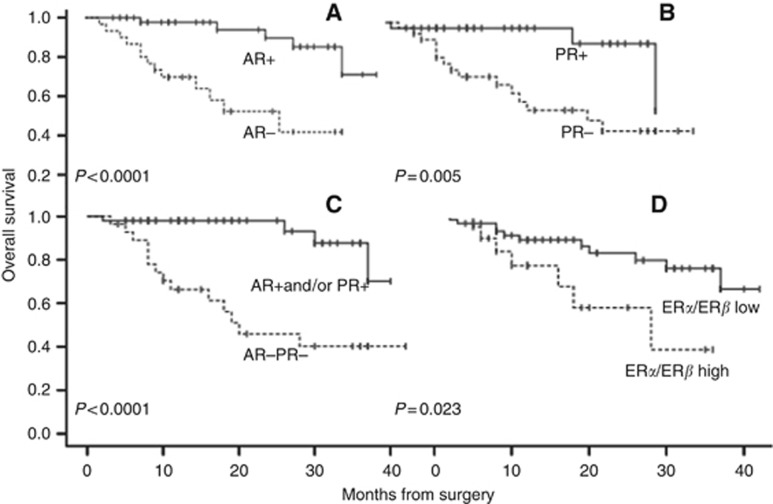
Kaplan–Meier survival curve for the probability of disease-free period relative to immunoexpression of (**A**) AR, (**B**) PR, (**C**) AR/PR negative and (**D**) ER*α*/ER*β* ratio.

Follow-up data were available for all EC patients. By January 2015 the median follow-up was 19 months, ranging between 6 and 40 months. During the follow-up period there were 5 recurrent tumours and 16 deaths (13 as a result of disease progression and 3 from other causes). ER*α* and ER*β* expression did not show a significant association with clinical outcome. A significant reduction in DFS was identified in the AR-negative group (*P*=0.0001) and PR-negative group (*P*=0.005). A subset of EC, which was negative for both AR and PR, showed a further decline in DFS (*P<*0.0001). Moreover, patients with a high ER*α*/*β* ratio had a worse prognosis (DFS, *P*=0.023) than those with a low ER*α*/*β* ratio (Figure 4). Univariate analysis has shown that HGEC, advanced stages (III and IV), deep myometrial invasion, cervical invasion, loss of AR, loss of PR, combined AR/PR loss and high ER*α*/ER*β* ratio were significantly associated with progressive disease (Table 2). None of the other parameters was significantly associated with patient outcome. Furthermore, Cox regression model confirmed that only AR (*P*=0.007), ER*α*/ER*β* (*P*=0.032), tumour grade (*P*=0.047) and myometrial invasion (*P*=0.008) were independent prognostic indicators (Table 2).

### Steroid receptor mRNA levels reflect their protein expression

Consistent with the IHC results, a general decline in mRNA levels for all steroid receptors was observed in HGEC. This reduction was significant for AR (*P*=0.0002) when compared with PM endometrium, and for ER*α* (*P*=0.003) when compared with LGEC. PR transcript level in HGEC was significantly lower than both PM (*P*=0.0002) and LGEC (*P*=0.001). The change in ER*β* mRNA was not significant (Figure 2F). Furthermore, AR (*r*=0.59, *P*=0.015) and PR (*r*=0.74, *P*=0.001) mRNA levels also showed a significant correlation with their protein expression scores (data not shown).

## Discussion

To our knowledge, this is the first comprehensive report comparing AR expression in healthy PM endometrium with healthy premenopausal PP endometrium and all EC subtypes, including non-endometrioid type II (serous and clear-cell carcinomas and carcinosarcoma) ECs and metastatic lesions. We have also described the contemporaneous expression scores for ER*α*, ER*β* and PR in serial sections of the same endometrial samples, allowing inferences to be made regarding their functional interplay in both healthy endometrium and in endometrial carcinogenesis.

Previous reports of steroid receptor protein expression in normal and pathological endometrium have used different semi-quantification methods (Mertens *et al*, 2001; Critchley *et al*, 2002; Mylonas *et al*, 2007; Zannoni *et al*, 2013). Of the available quickscores, Allred and IRS have been commonly used to analyse steroid receptors in endometrium (Mylonas *et al*, 2007; Zannoni *et al*, 2013), but these two systems were optimised for ER and PR expression (not AR) specifically in breast tissue. We propose a LESQS, which is optimised for both normal and neoplastic endometrium. Importantly, LESQS had the best correlation with a standard *H*-score for AR and ER*α* compared with both Allred and IRS scores for either epithelial or stromal compartments. LESQS also showed high correlation with the *H*-score for PR and ER*β*, similar to the Allred score correlation (Supplementary Table 6). In order to gain better insight into hormone actions, all cognate steroid receptors (AR, ER*α*, ER*β* and PR) need to be assessed using the same scoring system; hence the optimised LESQS was chosen.

Postmenopausal endometrium is composed of inactive glands lying in a compact stroma that morphologically resembles the stratum basalis of premenopausal endometrium (McCluggage, 2011). Moreover, PM endometrium is characterised by the complete loss of the stratum functionalis; therefore, we compared the steroid expression in PM endometrium with that of PP stratum basalis. Interestingly, compared with PP stratum basalis, we found significantly higher levels of epithelial AR and ER*α* in PM endometrium but similar ER*β* and PR expression levels. Horie *et al* (1992) reported AR expression (albeit in a sample size of *n*=4) in PM endometrial epithelium, which is consistent with our results, but they indicated the AR expression levels to be similar to PP stratum basalis. Their use of a small sample size without quantifying the immunostaining may explain the differences in conclusion between the two studies. In contrast, a previous report comparing PM endometrium with PP stratum functionalis suggested a decrease of ER*α*, ER*β* and PR expression in PM epithelial cells (Mylonas *et al*, 2007). The PM hormonal milieu is characterised by the absence of progesterone and oestradiol, the presence of low levels of circulating oestrone (Sivridis and Giatromanolaki, 2004) and persisting levels of androgens from the adrenals, which may support the maintenance of endometrial PR and ER expression. Although there are reports suggesting focal and weak epithelial AR expression appearing in late secretory phase endometrium, presumed to be associated with progesterone withdrawal (Horie *et al*, 1992; Mertens *et al*, 2001), the most prominent feature of premenopausal secretory phase endometrium is the high levels of stromal AR expression. This is in contrast with PM endometrium, where dominant AR staining is in the epithelium. Furthermore, anti-progesterone (mifepristone) administration was associated with upregulation of both stromal and epithelial AR expression in premenopausal primate endometrium (Slayden and Brenner, 2004), yet we observed the stromal AR to be low in PM compared with PP endometrium. As by definition, PM endometrium has not been exposed to progesterone for at least 12 months, the appearance of epithelial AR in PM endometrium cannot be equated merely to progesterone withdrawal. To complicate matters further, mifepristone is not only a progesterone antagonist but also has anti-androgenic and glucocorticoid properties (Hapangama, 2003). Therefore, the appearance of AR in PM epithelium may be induced by the action of either oestrone via ER*α* or androgens via AR (Fujimoto *et al*, 1994; Lovely *et al*, 2000). Interestingly, the upregulation of PM epithelial AR was not associated with high cell proliferation (assessed by Ki67), which would be expected to be present with classical ER*α*-mediated epithelial action. Short-term treatment with testosterone was associated with low PI in normal PM women (Zang *et al*, 2007). Consistent with *in vitro* studies (Tuckerman *et al*, 2000; Gibson *et al*, 2014), this indicates a direct androgen-driven induction of AR, resulting in an inhibition of PM epithelial proliferation. Taking in account that stromal AR has been described to have an antiapoptotic role (Marshall *et al*, 2011), AR seems to have a cell-specific function in the endometrium.

Endometrial hyperplasia with atypia is a premalignant condition (Lacey *et al*, 2008) with molecular aberrations (Steinbakk *et al*, 2011) and morphological changes that are typical for unopposed oestrogenic activity. Consistent with previous reports, the epithelial expression of AR in EHA was higher than that in PP endometrium (Ito *et al*, 2002), whereas the PI of EHA did not differ from that of PP endometrium. Intriguingly, compared with PM endometrium, AR scores were relatively lower in EHA, with a significantly high Ki67 confirming oestrogen-driven cell proliferation.

The available data on steroid receptor expression in EC subtypes are largely confined to endometrioid EC focusing on ER*α*, ER*β* and PR (Deligdisch *et al*, 2000; Kounelis *et al*, 2000; Collins *et al*, 2009). Traditionally, type II ECs are considered to be hormonally independent; there are only limited reports of ER and PR expression (Alkushi *et al*, 2010; Mhawech-Fauceglia *et al*, 2013), with no previous data on AR and ER*β* expression in serous and clear-cell carcinomas and carcinosarcomas (Hapangama *et al*, 2015). However, recent reports indicate that both type I and II ECs have similar risk factors (Setiawan *et al*, 2013). Studies describing AR expression in endometrioid EC are scarce and inconsistent. Horie *et al* (1992) reported positive AR expression in four grade 2 EC samples. In our study, most LGECs expressed AR (protein and transcript), and the LGEC immunoscores did not significantly differ from those of PM controls. Grade 3 endometrioid EC, however, expressed lower levels of AR consistent with the previous report (Ito *et al*, 2002). Conflicting reports have suggested that AR is absent in 72% ECs (Sasaki *et al*, 2000), although, in that particular study, the specific histological type was not described for all samples. In agreement with our results, others have reported downregulation of PR in non-endometrioid HGEC (Alkushi *et al*, 2010). Interestingly, in comparison with LGECs, HGEC showed a significant decline of both PR and AR protein expression, whereas the decrease in ER*α* was not significant. This was particularly evident in clear-cell carcinoma, although conflicting reports have shown a reduction of ER (without distinguishing ER*α* or ER*β* subtypes) in clear-cell EC (Hoang *et al*, 2013; Mhawech-Fauceglia *et al*, 2013). Differences in sample size and methodology (antibodies and immunoanalysis) are likely to be the explanation for this discrepancy. In agreement with our data, the recent TCGA data also suggest AR expression to be a feature of LGECs with better prognosis (Kandoth *et al*, 2013). Furthermore, it was interesting that although ESR1 (ER*α*) and PGR (PR) gene mutation was seen in endometrial cancers with favourable outcome, AR mutations were not identified in the large TCGA data set, suggesting the therapeutic potential of AR receptor modulators in EC.

From these results, it is tempting to speculate that even in presumed ‘hormonally independent' non-endometrioid HGEC, ER*α* and ER*β* receptors were expressed relatively abundantly (with concomitant loss of AR and PR), resulting in possible unopposed oestrogenic activity.

Our data examined AR expression in metastatic lesions for the first time, and demonstrated significantly higher AR expression than in the matched primary tumours. Although molecular and biological characteristics of macro-metastases are not well defined, endometrial metastatic epithelial cells have been reported to express oestrogen receptors, ER*β* in particular (Fujimoto *et al*, 2002). The metastatic lesions examined in the present study showed some similarities with the primary tumours such as the PR expression and PI. Sixty per cent of these lesions showed loss of PR, consistent with previous reports (Tangen *et al*, 2014) but interestingly, in these lesions, AR expression was significantly upregulated. Low PR expression may explain the poor clinical response observed in a majority (66%) of patients with recurrent or metastatic tumours to progesterone treatment (Fiorica *et al*, 2004). The re-emergence of AR expression in these lesions, however, produces a possible novel adjuvant therapeutic opportunity.

Unlike breast cancer, the literature on the prognostic value of ER*α*, ER*β*, ER*α* /ER*β* ratio and PR in EC is inconsistent, probably owing to methodological variations (Takama *et al*, 2001; Fujimoto *et al*, 2002; Shabani *et al*, 2007; Jongen *et al*, 2009; Zannoni *et al*, 2013). Our results have shown that AR and PR expression is associated with longer DFS in patients with EC, and this is consistent with other recent reports (Tanaka *et al*, 2014; Tangen *et al*, 2014), and concurrent loss of both AR and PR was associated with a higher risk of relapse. Moreover, loss of AR and PR correlated positively with unfavourable clinicopathological parameters that predict poor clinical outcome such as high-grade, deep myometrial invasion and cervical stromal involvement, and advanced FIGO stages. AR may also bind progesterone, which is the main hormonal therapy in EC, and subsequently may mediate inhibition of cell proliferation (Hackenberg and Schulz, 1996). Further, emerging evidence indicates that androgens, unlike progestin, may induce PR expression (Park *et al*, 2014). Therefore, besides the prognostic significance, expression data on AR and PR may be a useful clinically as a potential therapeutic target to tailor adjuvant hormonal therapy for intermediate and higher-risk EC. We have also shown a high ER*α*/ER*β* ratio to be significantly associated with shorter DFS, and this is likely to be due to a relative reduction in expression of the guardian protein, ER*β*, that counteracts mitogenic ER*α* activity (Hapangama *et al*, 2015). These data conflict with some previous reports that only examined endometrioid EC, using either mRNA levels or different quantification methods of IHC staining (Takama *et al*, 2001; Zannoni *et al*, 2013).

We acknowledge that this is an observational analytic study, which requires further functional and genetic investigations in the future to confirm the clinical relevance of these findings. Further work will also evaluate the potential therapeutic effects of selective AR modulators in the management of advanced EC. Further, we believe that the characterisation of metastatic lesions for steroid receptor expression in a larger sample series ideally before and after hormonal therapy may unveil the reasons behind the limited responsiveness of EC to hormonal therapy. Our LESQS scoring system will provide a standard and clinically applicable tool for researchers to undertake these important future studies.

## Conclusions

Healthy PM endometrial epithelial cells acquire AR compared with healthy premenopausal proliferative endometrium, whilst ER*α*, ER*β* and PR expression is maintained. Nuclear epithelial AR expression appears to be associated with an anti-proliferative phenotype suggesting a cell-type-specific function of androgen. In EC, loss of AR and PR with persistent ER*α* and ER*β* expression may promote the unrestrained growth and propagation observed in HGEC. Therefore, ER subtypes may potentially represent a therapeutic target in HGEC, whilst AR may be a clinically relevant prognostic indicator.

## Figures and Tables

**Table 1:** Demographic features of study groups

**Study groups**	**Number of cases**	**%**	**Age**^a^ **(years)**	**BMI**^b^ **(kg m**^**−2**^)
Proliferative phase	14		39 (30–47)	26.7 (17.5–45.5)
Postmenopausal	14		68.5 (57–79)	26.3 (22.7–35.8)
Endometrial hyperplasia	12		50 (37–67)	29.7 (34.3)
Without cytological atypia	4		55 (50–62)	25.75 (23.6–53.2)
With cytological atypia	8		52 (37–67)	34 (27.9–57.8)
Endometrial cancer	85		67 (41–89)	30 (20.2–54.4)
LGEC	37	43.5	63.5 (41–84)	30.8 (21.6–46.1)
Grade1 endometrioid	19	22.4	63 (46–84)	35.7 (21.6–46.1)
Grade2 endometrioid	18	21.2	63.5 (41–83)	29 (22.3–54.4)
HGEC	48	56.5	70 (51–89)	29.6 (20.2–54.4)
Grade3 endometrioid	15	17.6	69 (51–83)	26.7 (22.1–42.7)
Serous	7	8.2	68 (64–82)	29.5 (24.8–34.9)
Clear cell	12	14.1	69 (52–82)	29.9 (25.4–31.5)
Carcinosarcoma	14	16.5	54.5 (59–89)	28.6 (20.2–37.2)
Metastatic lesions	16		68 (41–89)	b

Abbreviations: BMI=body mass index; HGEC=high-grade endometrial carcinoma; LGEC=low-grade endometrial carcinoma.

a Data expressed as median (range).

b BMI data were available for only 60 cases.

**Table 2:** Univariate and multivariate analyses of disease-free survival

**Variables**	**Univariate**	**Multivariate**	
	***P***	***P***	**HR (95% CI)**
Age (years): ⩽65 *vs* >65	0.328	Removed	—
BMI^a^: ⩽30 *vs* >30	0.531	Removed	—
Tumour stage: I–II *vs* III–IV	0.029	0.579	—
Tumour grade: LG *vs* HG	0.005	0.046	5.5 (1.0–29.8)
Lymphovascular invasion: − *vs* +	0.097	Removed	—
Myometrial invasion : ⩽50% *vs* >50%	0.002	0.008	8.8 (1.8–44.2)
Cervical stromal invasion: – *vs* +	0.0001	0.073	—
Extra uterine invasion: −*vs* +	0.19	Removed	—
AR expression: – *vs* +	0.001	0.007	0.12 (0.3–0.5)
PR expression: – *vs* +	0.012	0.771	—
AR−PR− expression: – *vs* +	0.001	0.727	—
ER*α* expression: – *vs* +	0.547	Removed	—
ER*β* expression: – *vs* +	0.895	Removed	—
ER*α*/*β* ratio: ⩽1 *vs* >1	0.031	0.032	3.5 (1.1–11.3)

Abbreviations: AR=androgen receptor; BMI=body mass index; CI=confidence interval; ER=oestrogen receptor; HG=high grade; HR, hazard ratio; LG=low grade; PR=progesterone receptor.

a Data were available for 60 patients only.
