# Supplementary figures and images for: Androgen receptors are acquired by healthy postmenopausal endometrial epithelium and their subsequent loss in endometrial cancer is associated with poor survival
Source: Br J Cancer. 2016 Mar 1;114(6):688–96. doi: 10.1038/bjc.2016.16 (PMC4800292; doi:10.1038/bjc.2016.16)

## Slide 1
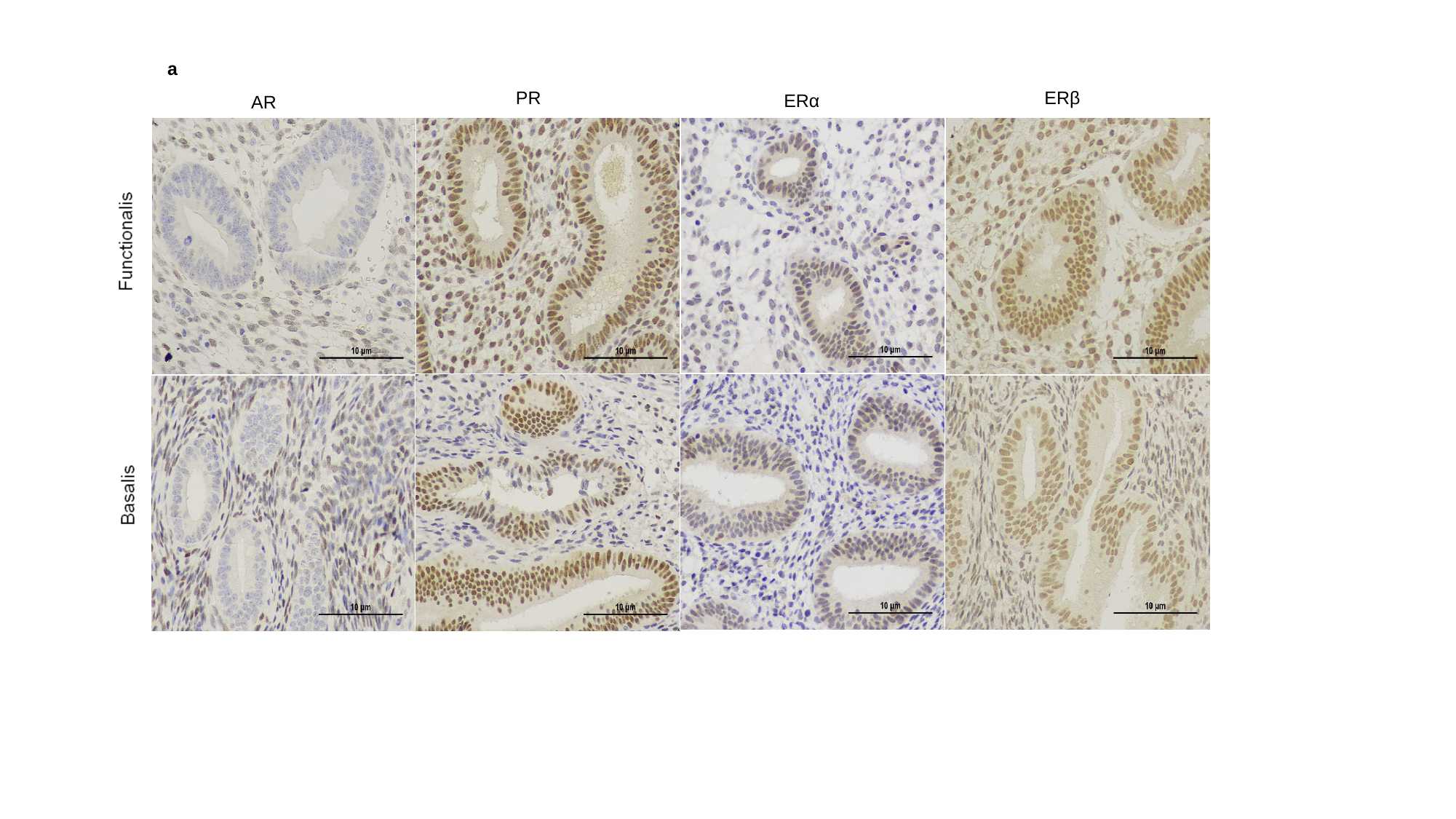

a
PR
ERβ
 ERα
AR

## Slide 2
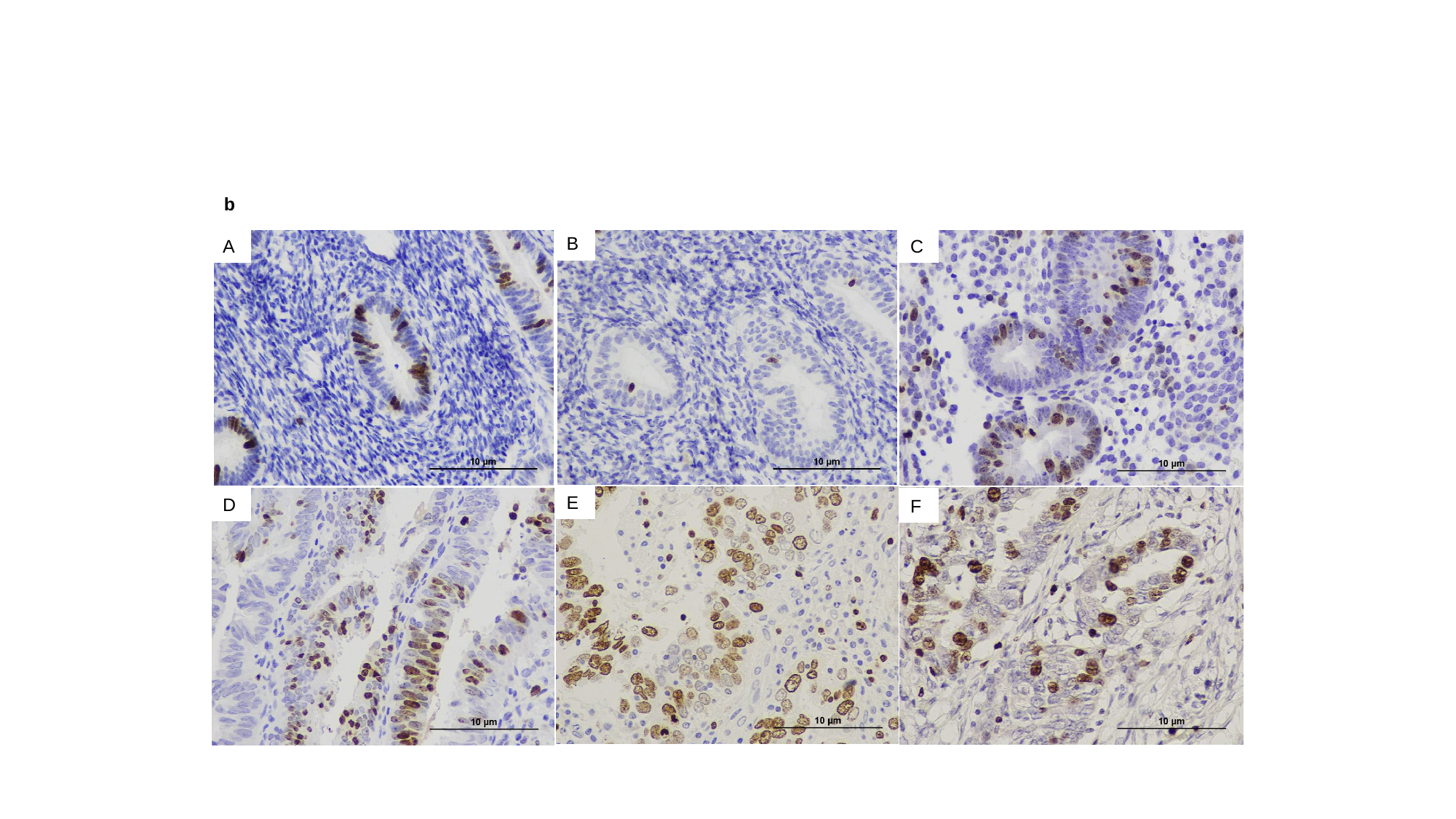

#
b
B
C
E
D
F
A

Supplement: Supplementary Figure 1 [file bjc201616x1.ppt]
