# Supplementary material for: Androgen receptors are acquired by healthy postmenopausal endometrial epithelium and their subsequent loss in endometrial cancer is associated with poor survival
Source: Br J Cancer. 2016 Mar 1;114(6):688–96. doi: 10.1038/bjc.2016.16 (PMC4800292; doi:10.1038/bjc.2016.16)
Supplement: Supplementary Figure Legend [file bjc201616x2.doc]

**Supplementary Figure 1** (a) Representative photomicrographs of immunolocaliztion of AR, PR, ERα and ERβ in basalis and functionalis layers of proliferative phase endometrium (b) Representative photomicrographs of immunoexpression of Ki67 in proliferative phase: A, postmenopausal: B, hyperplastic endometrium: C, low grade: D, high grade endometrial cancer: E, and metastatic lesion: F.
