# Supplementary material for: Androgen receptors are acquired by healthy postmenopausal endometrial epithelium and their subsequent loss in endometrial cancer is associated with poor survival
Source: Br J Cancer. 2016 Mar 1;114(6):688–96. doi: 10.1038/bjc.2016.16 (PMC4800292; doi:10.1038/bjc.2016.16)
Supplement: Supplementary Methods [file bjc201616x3.docx]

**Supplementary Methods**

Initially, we recorded the percentage of positively stained target cells in each of four intensity categories. We then calculated the H-score using the formula described by McCarty (1986):

Hscore = $\sum pi\left(i+1\right)$

where *i* represents the intensity of staining with a value of 0, 1, 2 or 3 (no staining, weak, moderate, strong staining) and *Pi*; varies from 0 to 100%. We then proposed a 4 tiered Liverpool endometrial steroid quick score (LESQS) based on the homogenous distribution of the observations in the arbitrary cut off categories within our endometrial samples (1%-10% =1, 11%-20% =2, 21%-40% =3, and >40%=4). The score for the proportion of positive cells was then multiplied by the staining intensity categories and added to give a final score out of 12. For example, if 40% of the cells were weakly positive (3x1=3), 20% moderately positive (2x2=4), 10% strongly positive (1x3=3) and the remaining 30% were negative (3x0=0), the total score will be: 3 + 4 + 3 + 0 = 10

We also employed two well established quick scores, the Allred score (17) and the immunoreactive score of Remmele and Stegner (IRS) (18) to identify the semi-quantitative scoring system that best reflects the steroid receptor profile in endometrium. The Allred score is a five-tier score rated as (≤ 1% =1, 1–10% =2, 11–33%=3, 34–66%=4 and 67–100% =5); the total score out of 8 is obtained by adding the score of the proportion with the predominant staining to the score of the intensity 0, 1, 2 or 3 (no staining, weak, moderate, strong staining). Therefore, for the example above, 40% (proportion score 4) of the sample expressed weak staining (intensity score 1) which is the predominant staining intensity; thus the total score is 4 + 1=5

The IRS score is a four-class score where 0 = no staining; 1 = ≤10% staining; 2 = 11–50% staining; 3 = 51–80% staining; and 4 = ≥81% staining. The total score out of 12 is obtained by multiplying the intensity score 0, 1, 2 or 3 by the proportion score. For the same example, (2x1) + (2x2) + (1x3) + (2x0) = 9

The LESQS, Allred score and IRS were correlated with the H score of corresponding samples in a subset of 37 EC samples which showed the lowest, intermediate and highest steroid expression levels. Compared with the H score, the LESQS showed the highest correlation for AR and ERα and good correlation for PR and ERβ (Supplementary Table 6). The LESQS was therefore preferentially chosen for analysis of expression of the four steroid hormone receptors in our samples.
